# Supplementary material for: Analysis of the genomic landscape of yolk sac tumors reveals mechanisms of evolution and chemoresistance
Source: Nat Commun. 2021 Jun 11;12:3579. doi: 10.1038/s41467-021-23681-0 (PMC8196104; doi:10.1038/s41467-021-23681-0)
Supplement: Supplementary file 1 — Supplementary Information [file 41467_2021_23681_MOESM1_ESM.pdf]

Supplement

Analysis of the Genomic Landscape of Yolk Sac Tumors Reveals Mechanisms of Evolution and Chemoresistance

Zong et al.

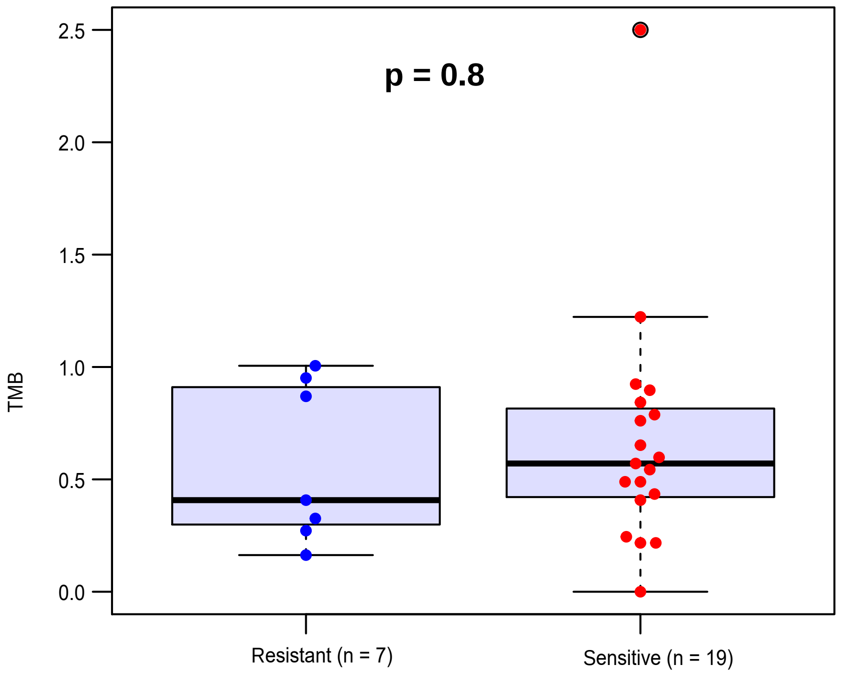

Supplementary Figure 1 | The TMB distribution of primary YST samples between resistant and sensitive groups. P value was calculated based on two-sided Wilcoxon rank-sum test (sensitive: n = 19 biologically independent samples; resistant: n = 7). The middle line in the box is the median, the bottom, and top of the box are the first and third quartiles, and the whiskers extend to 1.5× interquartile range of the lower and the upper quartiles, respectively.

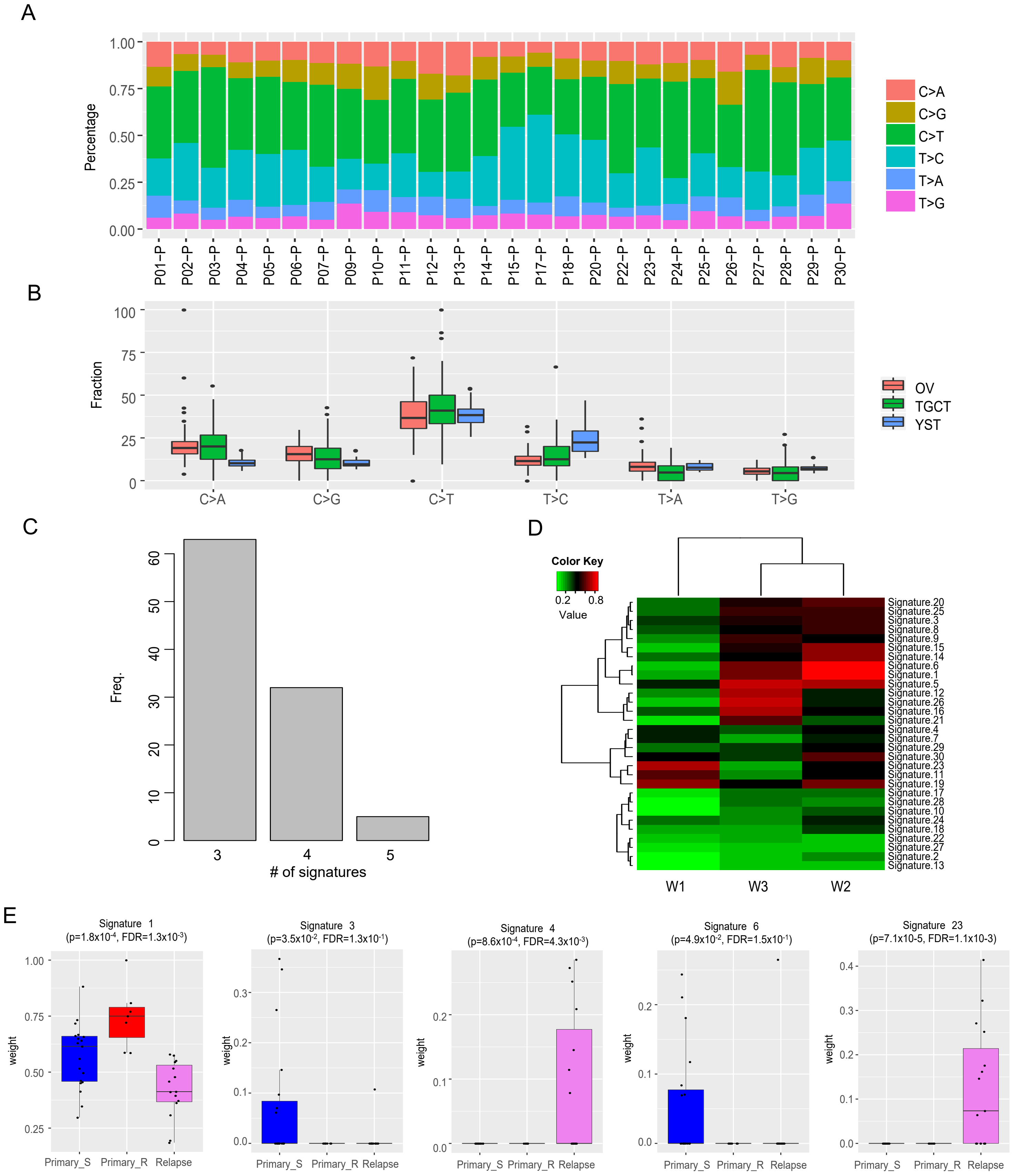

Supplementary Figure 2 | Mutation signatures of YSTs. (A) Mutation spectrum across all primary YST samples. Red indicates mutation type C>A/G>T, brown C>G/G>C, green C>T/G>A, light blue T>C/A>G, blue T>A/A>T, and purple T>G/A>C. (B) The distributions of six base substitutions among the primary tumor samples from ovarian cancer (OV:177), testicular germ cell tumors (TGCT: 144), and YST (26). (C) The frequencies of three, four and five signatures obtained through decomposing analysis; 100 independent analyses were performed. (D) The cosine similarity between signatures (W1, W2, and W3) identified in this study and those identified from the COSMIC database. (E) The comparison of known COSMIC signatures among chemo-sensitive primary, chemo-resistant primary, and relapsed samples. P values were computed based on Kruskal-Wallis tests, and FDR were used for multiple testing corrections. primary_sensitive: n = 19 biologically independent samples; primary_resistant: n = 7; relapse: n = 15. (B, E) In the box plots, the middle line in the box is the median, the bottom, and top of the box are the first and third quartiles, and the whiskers extend to 1.5× interquartile range of the lower and the upper quartiles, respectively.

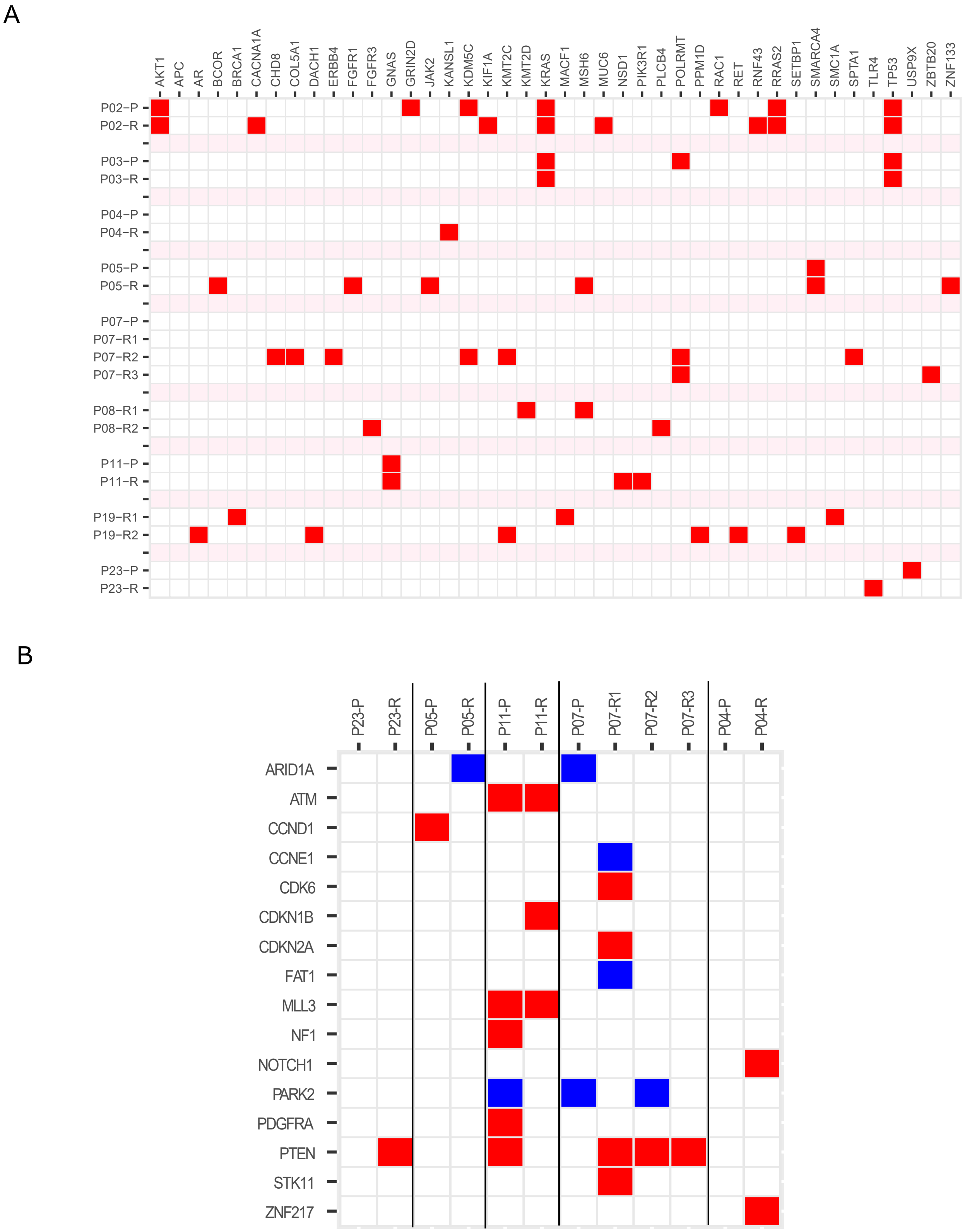

Supplementary Figure 3 | Somatic alterations of known cancer genes in primary and relapsed YSTs
A comparison of somatic mutations (A), and SCNAs (B) between primary and relapsed YSTs.

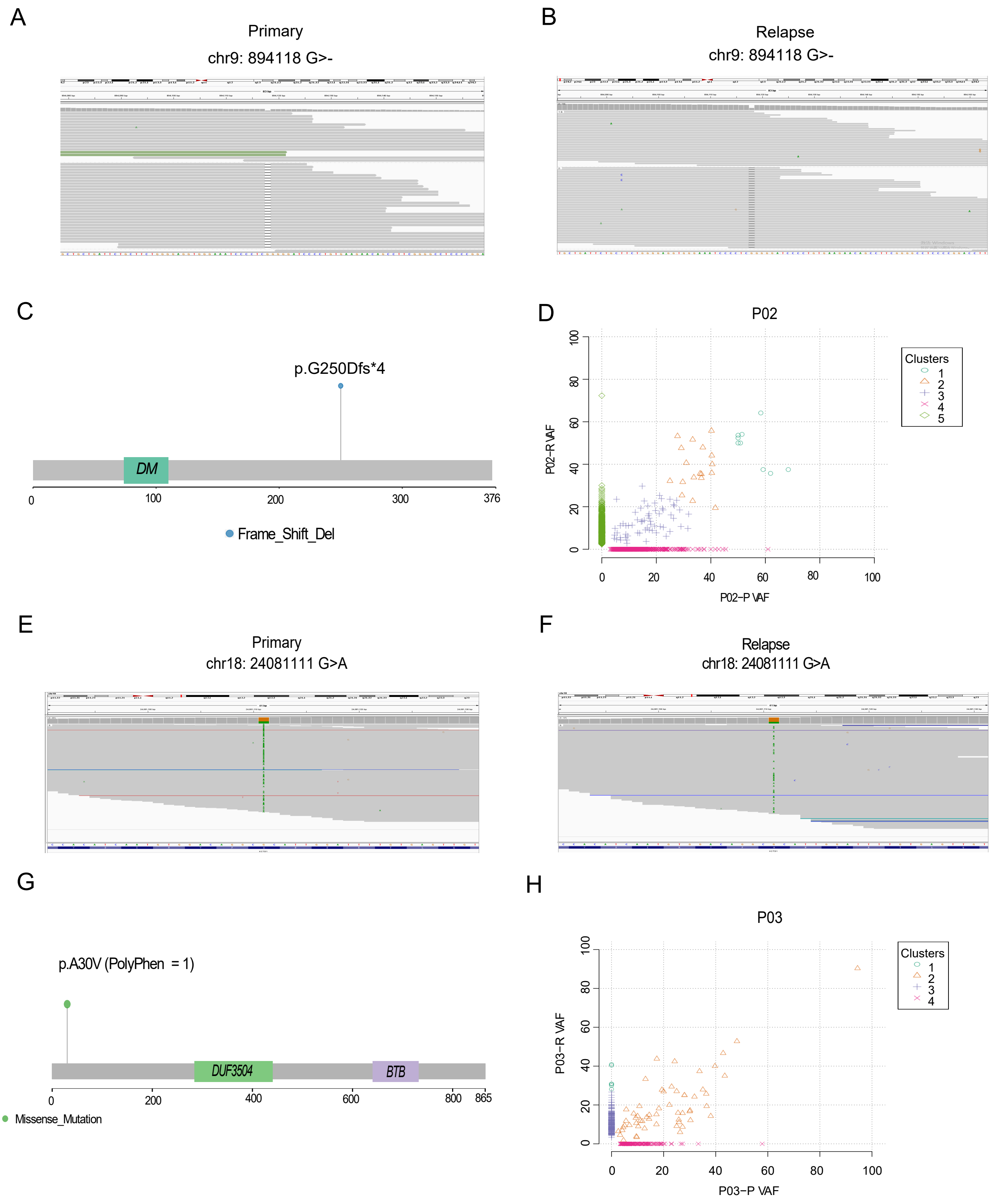

Supplementary Figure 4 | Loss-of-function mutation in DMRT1 and KCTD1 in the YST patients with gonadal dysgenesis. (A-B) DMRT1 frameshift mutation in primary (A) and relapse (B) samples from the patient P02. (C) The location of the amino acid mutation in DMRT1. (D) Mutation allele frequency of primary and relapsed tumor samples from P02. (E-F) KCTD1 missense mutation in primary (E) and relapse (F) tumor samples from patient P03. (G) The location of the amino acid mutation in KCTD1. (H) Mutation allele frequency of primary and relapsed tumor samples from P03. (D and H) Allele frequencies in primary (x-axis) and relapse (y-axis) paired samples in YST. Predicted clones or sub-clones is shown in specific shapes and color combinations.

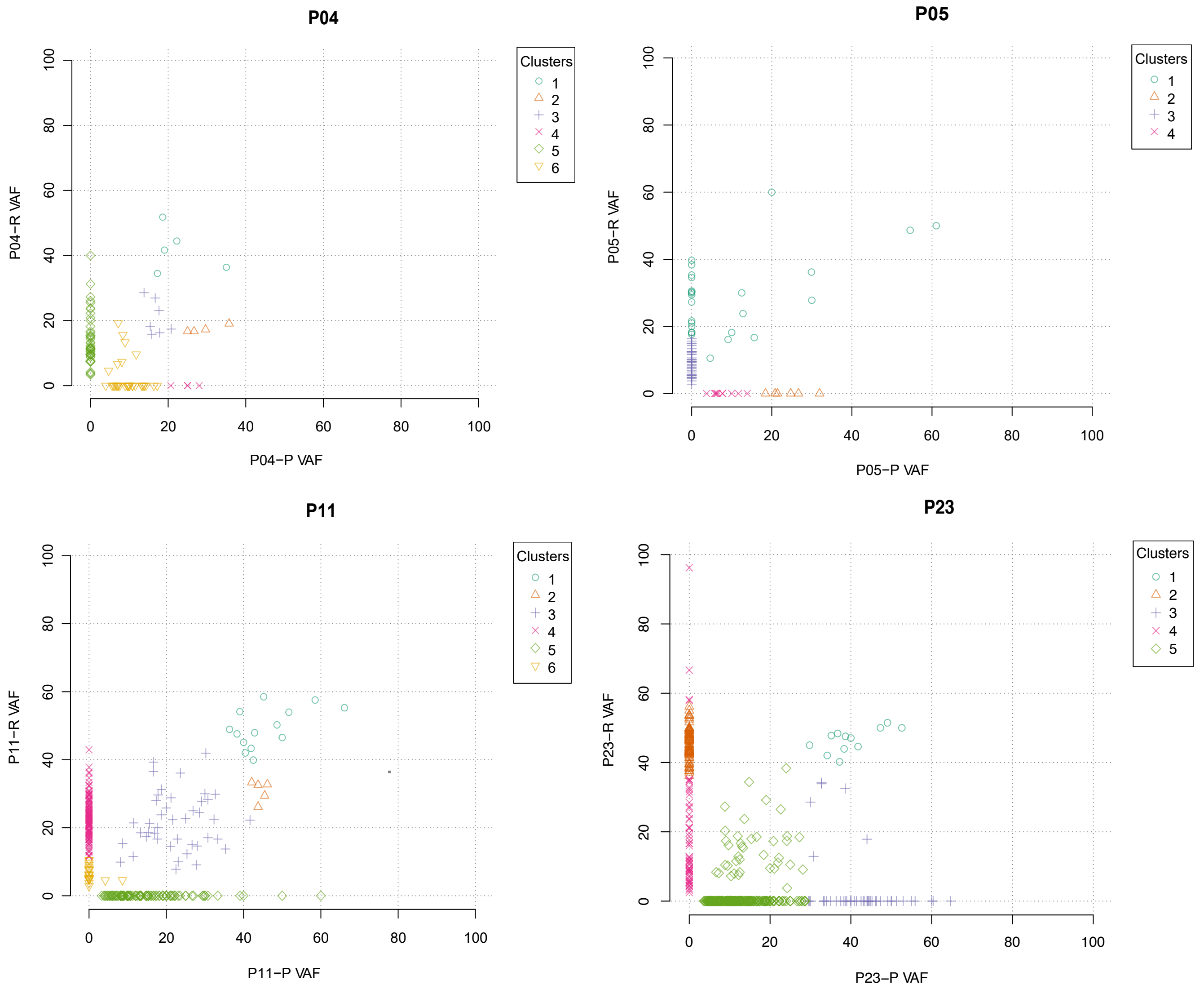

Supplementary Figure 5 | Mutation allele frequency of primary and relapsed tumors from P04, P05, P11, and P23. Allele frequencies in primary (x-axis) and relapse (y-axis) paired samples in YST. Predicted clones or sub-clones are shown in specific shapes and color combinations.

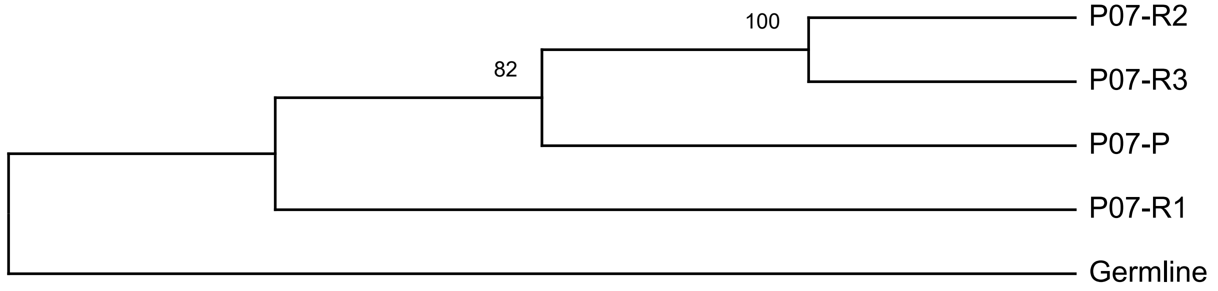

Supplementary Figure 6 | A phylogenetic tree showing evolutionary relationships between the tumor samples based on maximum parsimony. Numbers indicate the bootstrap values.

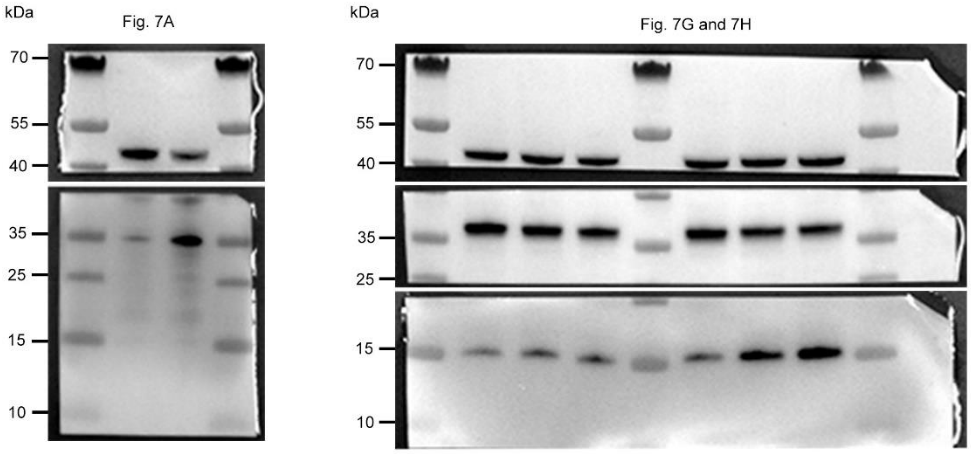

Supplementary Figure 7 | Full scans of Western blots shown in Figure 7

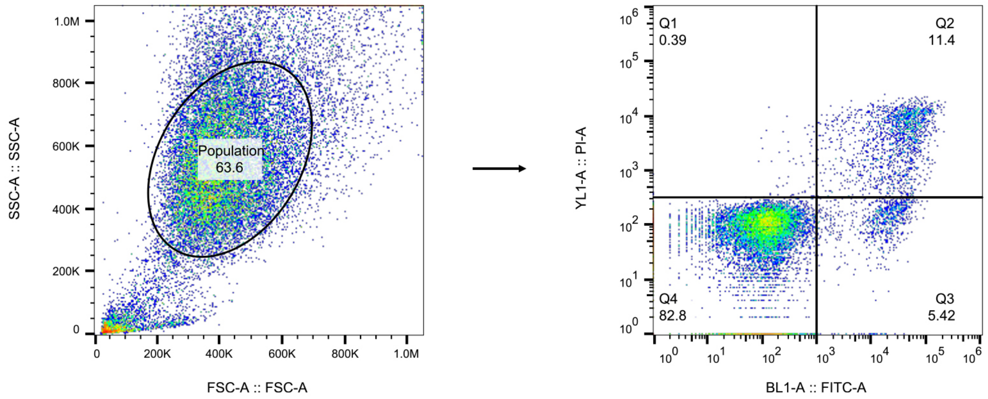

Supplementary Figure 8 | An example of gating strategy for flow cytometry analysis of apoptosis in NOY1 cells transfected with siRNAs.

Supplementary Table 1. significantly mutated genes identified by MuSiC2.

#Gene	Indels	SNVs	Tot Muts	Covd Bps	Muts pMbp	P-value CT	FDR CT
ZNF708	0	3	3	35497	84.51	6.42E-09	0.00013
C8orf44	0	2	2	20496	97.58	2.27E-06	0.01987
KIAA1549L	0	3	3	191592	15.66	3.89E-06	0.01987
SOX1	1	1	2	21915	91.26	3.00E-06	0.01987
HSPA6	2	0	2	48504	41.23	6.84E-06	0.02795
DYNC1I1	0	2	2	110616	18.08	2.50E-05	0.08255
FRG1	1	1	2	43426	46.06	2.83E-05	0.08255
SLC9A8	0	2	2	83145	24.05	4.30E-05	0.10976
NBPF10	0	4	4	1122700	3.56	5.97E-05	0.12941
KRAS	0	2	2	37052	53.98	6.97E-05	0.12941
KIT	1	1	2	121536	16.46	6.59E-05	0.12941
APOL6	0	2	2	36600	54.64	8.15E-05	0.13287
TMEM247	0	2	2	28565	70.02	8.45E-05	0.13287
SLC46A2	0	2	2	47712	41.92	0.000111112	0.15443
NPAS1	1	1	2	85848	23.3	0.000113344	0.15443
C8orf33	0	1	1	27912	35.83	0.000135667	0.1733
