# Supplementary material for: Analysis of the genomic landscape of yolk sac tumors reveals mechanisms of evolution and chemoresistance
Source: Nat Commun. 2021 Jun 11;12:3579. doi: 10.1038/s41467-021-23681-0 (PMC8196104; doi:10.1038/s41467-021-23681-0)
Supplement: Supplementary file 3 — Description of Additional Supplementary Files [file 41467_2021_23681_MOESM3_ESM.pdf]

Description of Additional Supplementary Files

File Name: Supplementary Data 1
Description: The clinical and sequencing information of tumor samples from YST patients in this study.

File Name: Supplementary Data 2
Description: Somatic mutations identified in this study.
