# Supplementary material for: Analysis of the genomic landscape of yolk sac tumors reveals mechanisms of evolution and chemoresistance
Source: Nat Commun. 2021 Jun 11;12:3579. doi: 10.1038/s41467-021-23681-0 (PMC8196104; doi:10.1038/s41467-021-23681-0)
Supplement: Supplementary file 6 — Reporting Summary [file 41467_2021_23681_MOESM6_ESM.pdf]

nature research

Corresponding author(s): Liang

Last updated by author(s): Sep 30, 2020

Reporting Summary

Nature Research wishes to improve the reproducibility of the work that we publish. This form provides structure for consistency and transparency in reporting. For further information on Nature Research policies, see our Editorial Policies and the Editorial Policy Checklist.

Statistics

For all statistical analyses, confirm that the following items are present in the figure legend, table legend, main text, or Methods section.

n/a	Confirmed	
☐	☒	The exact sample size (*n*) for each experimental group/condition, given as a discrete number and unit of measurement
☐	☒	A statement on whether measurements were taken from distinct samples or whether the same sample was measured repeatedly
☐	☒	The statistical test(s) used AND whether they are one- or two-sided<br>*Only common tests should be described solely by name; describe more complex techniques in the Methods section.*
☐	☒	A description of all covariates tested
☐	☒	A description of any assumptions or corrections, such as tests of normality and adjustment for multiple comparisons
☐	☒	A full description of the statistical parameters including central tendency (e.g. means) or other basic estimates (e.g. regression coefficient) AND variation (e.g. standard deviation) or associated estimates of uncertainty (e.g. confidence intervals)
☐	☒	For null hypothesis testing, the test statistic (e.g. *F*, *t*, *r*) with confidence intervals, effect sizes, degrees of freedom and *P* value noted<br>*Give P values as exact values whenever suitable.*
☒	☐	For Bayesian analysis, information on the choice of priors and Markov chain Monte Carlo settings
☒	☐	For hierarchical and complex designs, identification of the appropriate level for tests and full reporting of outcomes
☐	☒	Estimates of effect sizes (e.g. Cohen's *d*, Pearson's *r*), indicating how they were calculated

Our web collection on statistics for biologists contains articles on many of the points above.

Software and code

Policy information about availability of computer code

Data collection	Burrows-Wheeler-Aligner v0.7.17; Genome Analysis Toolkit v3.8.1; BAM-matcher; SignatureAnalyzer; MANTIS v1.0.3; MuSiC2 v0.2; CNVkit v0.9.3; sciClone v1.10; MEGAX; clusterProfiler v3.14.3
Data analysis	The analyses were conducted using R v3.6.2.

For manuscripts utilizing custom algorithms or software that are central to the research but not yet described in published literature, software must be made available to editors and reviewers. We strongly encourage code deposition in a community repository (e.g. GitHub). See the Nature Research guidelines for submitting code & software for further information.

Data

Policy information about availability of data

All manuscripts must include a data availability statement. This statement should provide the following information, where applicable:

- Accession codes, unique identifiers, or web links for publicly available datasets
- A list of figures that have associated raw data
- A description of any restrictions on data availability

The raw sequence data reported in this study have been deposited in the Genome Sequence Archive of the BIG Data Center, Beijing Institute of Genomics (BIG), Chinese Academy of Sciences, under accession number HRA000131 [http://bigd.big.ac.cn/gsa].

Field-specific reporting

Please select the one below that is the best fit for your research. If you are not sure, read the appropriate sections before making your selection.

☒ Life sciences ☐ Behavioural & social sciences ☐ Ecological, evolutionary & environmental sciences

For a reference copy of the document with all sections, see nature.com/documents/nr-reporting-summary-flat.pdf

Life sciences study design

All studies must disclose on these points even when the disclosure is negative.

Sample size	Sample size = 30
Data exclusions	No data exclusion
Replication	Each tumor sample is independent. For cell line studies, each experiment was replicated for at least 3 times.
Randomization	NA
Blinding	NA

Reporting for specific materials, systems and methods

We require information from authors about some types of materials, experimental systems and methods used in many studies. Here, indicate whether each material, system or method listed is relevant to your study. If you are not sure if a list item applies to your research, read the appropriate section before selecting a response.

Materials & experimental systems

n/a	Involved in the study	
☐	☒	Antibodies
☐	☒	Eukaryotic cell lines
☒	☐	Palaeontology and archaeology
☒	☐	Animals and other organisms
☐	☒	Human research participants
☐	☒	Clinical data
☒	☐	Dual use research of concern

Methods

n/a	Involved in the study	
☒	☐	ChIP-seq
☐	☒	Flow cytometry
☒	☐	MRI-based neuroimaging

Antibodies

Antibodies used	Anti-Caspase-3 antibody (Abcam, ab32351), Anti-Cleaved Caspase-3 antibody (Abcam, ab32042), Anti-OVOL2 antibody (Abcam, ab169469), Anti-beta actin antibody (Abcam, ab8226)
Validation	Anti-Caspase-3 antibody is rabbit monoclonal to Caspase-3 and specific for the pro form of human Caspase-3. This product can be applied in Western-blot and detects a band of approximately 35 kDa.<br>Anti-Cleaved Caspase-3 antibody is rabbit monoclonal to Cleaved Caspase-3 and specific for human Cleaved Caspase-3. This product can be applied in Western-blot and detects a band of approximately 17 kDa.<br>Anti-OVOL2 antibody is mouse polyclonal to OVOL2 and specific for human OVOL2. This product can be applied in Western-blot and detects a band of approximately 30 kDa.<br>Anti-beta Actin antibody is mouse monoclonal to beta Actin-Loading Control. This product can be applied in Western-blot and detects a band of approximately 42 kDa.

Eukaryotic cell lines

Policy information about cell lines

Cell line source(s)	NOY1 was purchased from a laboratory at Nagoya University. HOSEpiC was a gift from Dr. Keng Shen who bought this cell line from Peking Union Medical College.
Authentication	The NOY1 cell line originated from the yolk sac tumor of a 28-year-old female. The NOY1 cell lin has been shown to form tumors in vivo, nude mice injected with NOY1 cells developed peritoneal disseminations, which stained positive for AFP via immunohistochemistry.<br>HOSEpiC was isolated from human ovary. HOSEpiC are characterized by immunofluorescence with antibodies specific to cytokeratin-14, cytokeratin-18, and/or cytokeratin-19.

Mycoplasma contamination	The cell lines were tested negative for mycoplasma contamination.
Commonly misidentified lines (See ICLAC register)	None

Human research participants

Policy information about studies involving human research participants

Population characteristics	The 30 YST patients in this study were recruited at Peking Union Medical College Hospital, China
Recruitment	The patients were treated at Peking Union Medical College Hospital, and the tumor samples were collected after being evaluated as yolk sac tumor by pathologists.
Ethics oversight	Ethics Committee of Peking Union Medical College Hospital, Beijing (project ID: JS-1747)

Note that full information on the approval of the study protocol must also be provided in the manuscript.

Clinical data

Policy information about clinical studies

All manuscripts should comply with the ICMJE guidelines for publication of clinical research and a completed CONSORT checklist must be included with all submissions.

Clinical trial registration	*Provide the trial registration number from ClinicalTrials.gov or an equivalent agency.*
Study protocol	*Note where the full trial protocol can be accessed OR if not available, explain why.*
Data collection	*Describe the settings and locales of data collection, noting the time periods of recruitment and data collection.*
Outcomes	*Describe how you pre-defined primary and secondary outcome measures and how you assessed these measures.*

Flow Cytometry

Plots

Confirm that:

☒ The axis labels state the marker and fluorochrome used (e.g. CD4-FITC).

☒ The axis scales are clearly visible. Include numbers along axes only for bottom left plot of group (a 'group' is an analysis of identical markers).

☒ All plots are contour plots with outliers or pseudocolor plots.

☒ A numerical value for number of cells or percentage (with statistics) is provided.

Methodology

Sample preparation	NOY1 cells were transfected with the siRNAs for 48h and further treated with 80 μM cisplatin. At 24h, 48h after cisplatin treatment, NOY1 cells were harvested using trypsin digestion solution without EDTA (Solarbio, China) and washed with PBS twice. And then the cells were resuspended in binding buffer and incubated in the dark with FITC Annexin-V and propidiom iodide (Dojindo, Japan) for 15 min at room temperature.
Instrument	Attune NxT flow cytometer (Thermo Fisher Scientific, USA)
Software	FlowJo 10 software
Cell population abundance	*Describe the abundance of the relevant cell populations within post-sort fractions, providing details on the purity of the samples and how it was determined.*
Gating strategy	In FSC/SSC plot, cells were distributed diagonally and two distinct cell populations can be identified. The bottom left one was considered as cell debris, and the other one was the starting cell population.

☒ Tick this box to confirm that a figure exemplifying the gating strategy is provided in the Supplementary Information.
